# Initiation of wound healing is regulated by the convergence of mechanical and epigenetic cues

**DOI:** 10.1371/journal.pbio.3001777

**Published:** 2022-09-16

**Authors:** Tanay Bhatt, Rakesh Dey, Akshay Hegde, Alhad Ashok Ketkar, Ajai J. Pulianmackal, Ashim P. Deb, Shravanti Rampalli, Colin Jamora

**Affiliations:** 1 IFOM-inStem Joint Research Laboratory, Center for Inflammation and Tissue Homeostasis, Institute for Stem Cell Science and Regenerative Medicine, Bangalore, India; 2 National Centre for Biological Sciences, Bangalore, India; 3 School of Chemical and Biotechnology, SASTRA University, Thanjavur, Tamil Nadu, India; 4 Center for Inflammation and Tissue Homeostasis, Institute for Stem Cell Science and Regenerative Medicine, Bangalore, India; Harvard Medical School, UNITED STATES

## Abstract

Wound healing in the skin is a complex physiological process that is a product of a cell state transition from homeostasis to repair. Mechanical cues are increasingly being recognized as important regulators of cellular reprogramming, but the mechanism by which it is translated to changes in gene expression and ultimately cellular behavior remains largely a mystery. To probe the molecular underpinnings of this phenomenon further, we used the down-regulation of caspase-8 as a biomarker of a cell entering the wound healing program. We found that the wound-induced release of tension within the epidermis leads to the alteration of gene expression via the nuclear translocation of the DNA methyltransferase 3A (DNMT3a). This enzyme then methylates promoters of genes that are known to be down-regulated in response to wound stimuli as well as potentially novel players in the repair program. Overall, these findings illuminate the convergence of mechanical and epigenetic signaling modules that are important regulators of the transcriptome landscape required to initiate the tissue repair process in the differentiated layers of the epidermis.

## Abstract 2

Wound healing in the skin is a complex physiological process that entails a cell state transition from homeostasis to repair. This study reveals a mechanism involving nuclear translocation of DNA methyltransferase 3A (DNMT3a) that initiates the wound-healing process and is perturbed in skin diseases such as psoriasis.

## Introduction

The wound healing program in an epithelial tissue is fundamentally a product of cell state transitions from homeostasis to a repair program. In particular, cutaneous wound healing in the adult is an intricately regulated system wherein keratinocytes and many other cell lineages exhibit their plasticity as they undergo reprogramming, to carry out otherwise dormant functions, to rebuild the damaged skin. Many of the phenomena that occur in the repair process in adult skin are, in fact, reminiscent of cellular events that operate during fetal development [1]. At the other extreme, inappropriate activation of these repair processes can manifest as tissue pathology, which forms the foundation of the perception of diseases with a “wound signature” [2]. The question that arises is how the whole scale changes in gene expression are accomplished in order to facilitate this cellular reprogramming.

Recently, epigenetic regulators have emerged as a vital component capable of transiently rewiring the cell’s transcriptional program to mediate the continual regeneration of the mouse epidermis [3,4]. This mode of gene regulation operates at multiple levels ranging from histone and DNA modifications, chromatin remodeling, and activity of various subtypes of RNA species such as non-coding RNAs and micro-RNAs (miRNAs) [5,6]. These epigenetic mechanisms can thus have a profound impact on the transcriptional landscape of the cell and can easily be envisioned to participate in the transient activation or repression of approximately 1,000 genes that are required for wound closure [7]. Circumstantial evidence in support of a role for epigenetics in tissue repair comes from reports of the dynamic expression of several epigenetic regulators following injury to the skin. For instance, Ezh2, Suz12, and Eed, which are components of the polycomb repressive complex 2 (PRC2), are down-regulated, whereas the histone methylases JMJD3 and Utx are up-regulated upon tissue damage and all return to homeostatic levels upon the completion of wound closure [8]. While the description of various epigenetic players in epidermal homeostasis and wound healing are reported, the identity and function of their upstream regulators are, to a large extent, absent in the literature.

An intriguing candidate for an upstream regulator in a highly tensile tissue such as the epidermis are mechanical cues. The epidermis is a stratified epithelium comprised of a basal layer of proliferation competent keratinocytes and suprabasal layers of differentiated cells glued together via intercellular adhesion complexes that partly endows the tissue with its barrier function. In different cell types, changes in mechanical tension have been documented to induce the nuclear translocation of important transcription factors—a notable example of which is YAP/TAZ that has proliferation stimulating gene targets [9]. Many studies, including those on epidermal homeostasis and wound healing, have primarily focused on the changes in gene expression in proliferating cells [10,11]. On the other hand, differentiated cells, such as the suprabasal keratinocytes near the surface of the epidermis, have largely been relegated to bystander status. In spite of this, a few reports suggest that these neglected pools of differentiated cells are not inert in the cellular crosstalk that mediates the early responses of the tissue to injury. In particular, the uppermost layer of differentiated keratinocytes in the epidermis expresses caspase-8 that has a non-canonical role in regulating the wound healing program. We previously demonstrated that the down-regulation of caspase-8 is a natural phenomenon upon application of an excisional wound to the mouse skin [12]. This down-regulation is particularly relevant as genetically ablating caspase-8 in the epidermis is sufficient to induce a wound healing response even in the absence of any damage to the organ. In addition, the down-regulation of caspase-8 in the upper, differentiated layer of the epidermis mediates signaling networks to incite epithelial stem cell proliferation in the epidermis [12] and the hair follicle [13,14] to fuel wound closure. We have thus used the down-regulation of caspase-8 as a cellular biomarker to identify the higher order regulatory machinery that reprograms the cell to enter the wound healing process in differentiated keratinocytes, which are emerging as an important participant in the tissue repair program.

## Results

### Wound induced down-regulation of caspase-8 RNA correlates with the degree of promoter methylation

Previously, we have established the importance of the down-regulation of caspase-8 RNA in both physiological (wound healing [12]) as well as pathological (atopic dermatitis [15] and psoriasis [16]) scenarios. The mechanisms responsible for this down-regulation, however, remain unknown. Uncovering the regulatory machinery of caspase-8 RNA also holds the promise of understanding the process by which cells transition from a state of homeostasis to repair. Moreover, it can provide potential new therapeutic targets for common inflammatory skin diseases where this regulation is perturbed.

**Fig 1:**
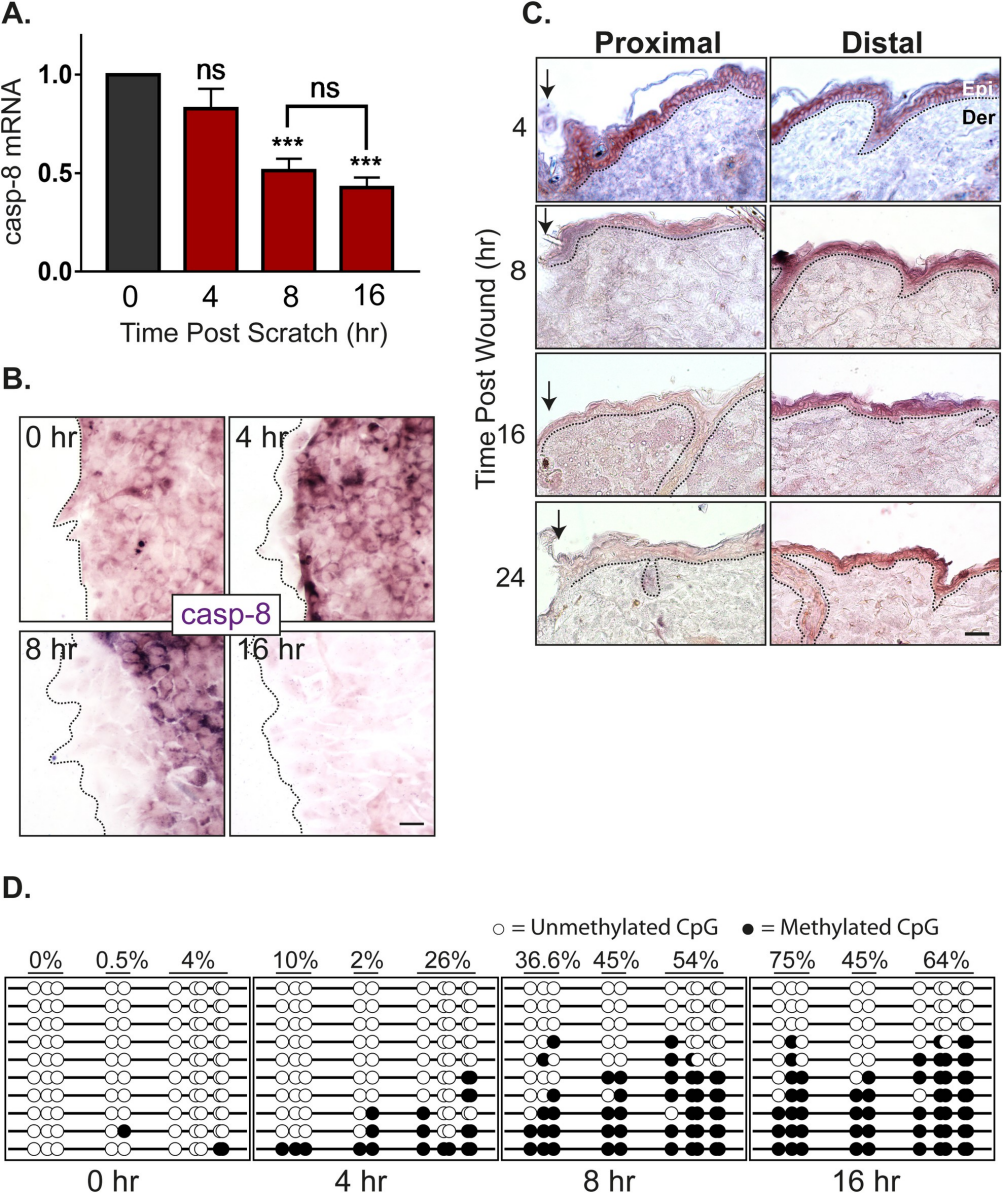
Kinetics of caspase-8 promoter methylation and expression. (**A**) Levels of caspase-8 mRNA at different time points post-scratch wound (fold change) (*n* = 4). (**B**) In vitro ISH of caspase-8 mRNA showing its levels at scratch margins over time [scale = 10 μm]. (**C**) In vivo ISH of caspase-8 mRNA showing its levels at wound proximal and distal regions over time (dotted line represents basement membrane, Epi = Epidermis, Der = Dermis) [scale = 20 μm]. (**D**) Bisulphite sequencing of caspase-8 promoter proximal region (265 bp) shows methylation status of 10 individual CpG sites (columns) from 10 cloned PCR products (rows) at various time points post-scratch wound. Percentage value denotes the percent methylation for each group of CpG sites over time (refer S1D Fig for the sequenced region and primer sites, *n* = 5 with 2 technical replicates). (Data are shown as mean ± SEM, *P*-values were calculated using 1-way ANOVA with Dunnett’s test and 2-tailed *t* test (A), *** *P* ≤ 0.001, ns = *P* > 0.05). Data underlying the graphs can be found in Fig 1A of S1 Raw Data.

RNA down-regulation can be achieved either via blocking the synthesis and/or active degradation. In order to distinguish between these 2 possibilities, we determined the half-life of caspase-8 in homeostasis compared to wound conditions. In differentiated primary epidermal keratinocytes, we observed that the half-life of caspase-8 mRNA under homeostatic conditions in vitro is approximately 2 hours (S1A Fig). In an in vitro scratch wound assay with multiple scratches, the level of caspase-8 RNA is significantly reduced by 8 hours (Fig 1A). Since the reduction of caspase-8 is faster under homeostatic conditions compared to the wound healing context, merely blocking RNA synthesis can achieve the reduction of caspase-8 mRNA and initiate the downstream wound healing response. Interestingly, the reduction caspase-8 RNA is localized in cells near the front of the scratch wound in vitro (Figs 1B and S1B). In situ hybridization of caspase-8 RNA demonstrates that the down-regulation can clearly be visualized in the cells immediately adjacent to the leading edge of a single scratch wound as early as 4 hours post wounding. By 8 hours post wounding, the caspase-8 RNA is down-regulated in about 3 to 4 cell rows from the wound front. These findings are consistent with our observation in excisional wounds on the back skin of mice where the decrease of caspase-8 RNA is visible as early as 4 hours in the wound proximal region (Figs 1C and S1C). Together, these results suggest that simply blocking transcription post injury is sufficient to down-regulate caspase-8. We hypothesized that the block in caspase-8 RNA synthesis is achieved through promoter methylation, which is consistent with previous reports documenting the same phenomenon in a variety of cancer cells through the hypermethylation of regulatory DNA sequence [17,18]. To understand whether this process in cancer cells is an aberration of the physiological healing program, we have assessed the methylation status of important regulatory sequences in the caspase-8 promoter, namely the CpG loci and SP1 binding sites (S1D Fig) [19]. Analysis of methylation of SP1 sites and other CpG loci reveals a time-dependent increase of promoter methylation in a sheet of differentiated epidermal keratinocytes subjected to multiple scratch wounds (Fig 1D). This progressive increase in the methylation of the caspase-8 promoter correlates well with the kinetics of the decrease in caspase-8 RNA (Fig 1A–1C). This suggests DNA methylation may play a critical role in regulating the wound healing response.

### Wound stimuli induce the nuclear localization of the DNA methyltransferase DNMT3a

**Fig 2:**
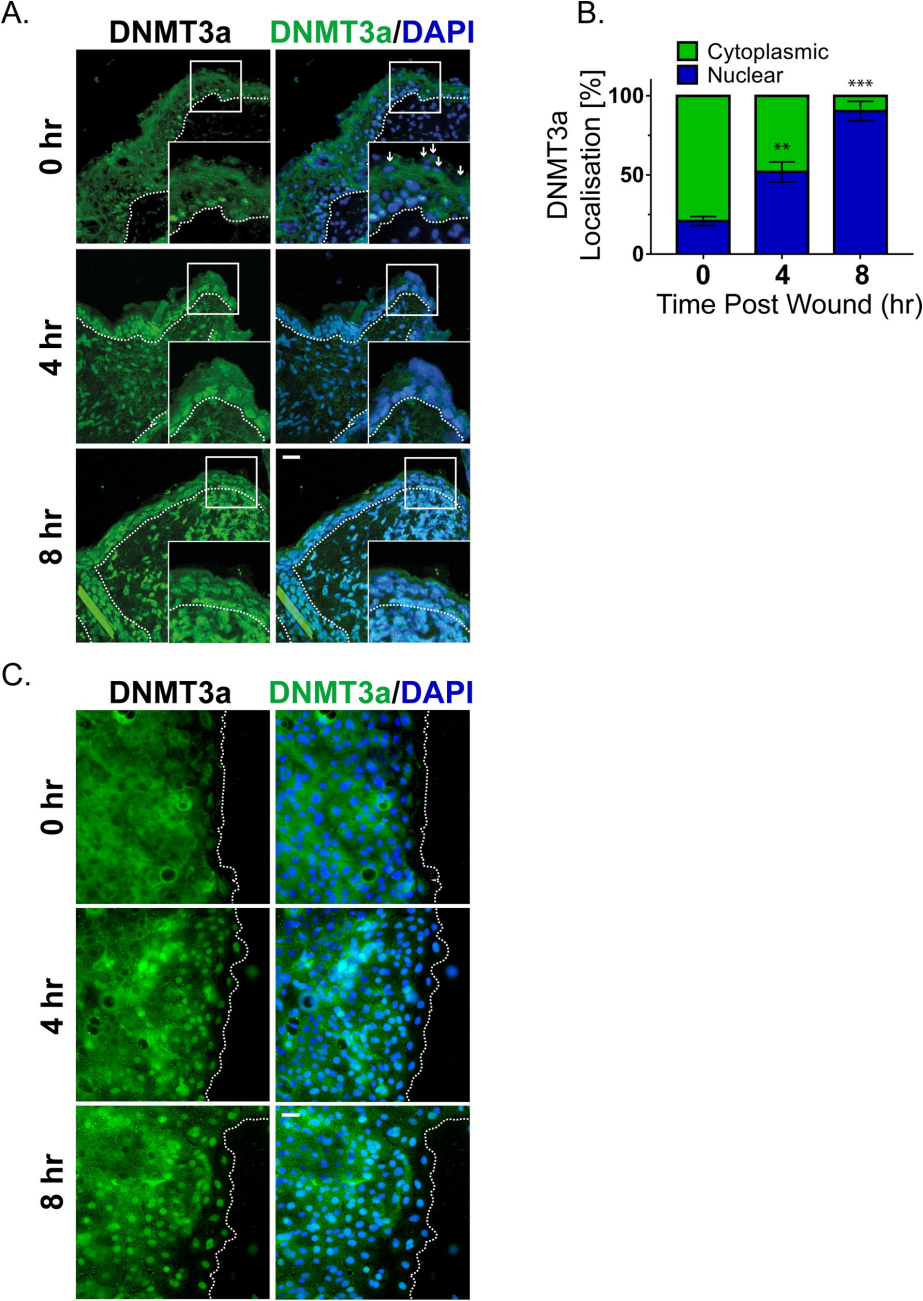
De novo DNMT3a increases nuclear localization at wound proximal site. (**A**) DNMT3a and DAPI staining of wound proximal (<0.5 mm) skin sections at different time interval (small white arrows showing nuclei of suprabasal keratinocytes, negative for DNMT3a staining). (**B**) Quantification and kinetics of DNMT3a localization (nuclear v/s cytoplasmic) from wound proximal (≤100 mm) skin sections. (It represents quantification of differentiated keratinocytes from the skin sections of 3 separate biological replicates.) (**C**) DNMT3a and DAPI staining of scratch wounded in vitro differentiated keratinocyte layer [scale = 20 μm]. (Images are representative of 3 biological replicates.) *P*-values were calculated using 1-way ANOVA with Dunnett’s test (B), *** *P* ≤ 0.001, ** *P*≤ 0.01, ns = *P* > 0.05. Data underlying the graphs can be found in Fig 2B of S1 Raw Data. DNMT3a, DNA methyltransferase 3A.

We thus investigated the mechanism responsible for DNA methylation of the caspase-8 promoter in response to injury. The bisulfite sequencing data reveals that the methylation of the caspase-8 promoter is a de novo event in response to wounding. We therefore examined the status of the 2 known de novo DNA methyltransferases (DNMTs), namely DNMT3a and DNMT3b, in response to injury. Interestingly, de novo DNMTs (DNMT3a and 3b) have also been shown to be important in regulating epidermal stem cell homeostasis [4]. To investigate whether these enzymes likewise play a role in tissue repair, we examined their expression in the wounded epidermis. Consistent with a previous report, under homeostatic conditions, we found that DNMT3a mainly resides in the nucleus of the basal/proliferating (K5 positive) cells and is absent or cytoplasmic in the suprabasal/differentiated (K5 negative) keratinocytes (S2A and S2B Fig) [20]. This localization was also recapitulated in vitro wherein we observed the cytosolic localization of DNMT3a in differentiated primary epidermal keratinocytes (S2C Fig). Interestingly, in vivo we observed that DNMT3a undergoes cytoplasmic to nuclear translocation in cells adjacent to the wound (Fig 2A). Quantification of the nuclear versus cytoplasmic localization of DNMT3a revealed a time-dependent accumulation of the enzyme in the nucleus post wounding (Fig 2B). This phenomenon was more apparent in an in vitro scratch assay, where keratinocytes adjacent to the scratch exhibited nuclear localization of DNMT3a (Fig 2C). The second known de novo DNMT, DNMT3b, also showed cytoplasmic localization in differentiated keratinocytes (S2D Fig). However, it did not translocate to the nuclei of wound proximal keratinocytes (S2E Fig).

Thus, we focused on understanding the mechanistic details of DNMT3a’s role in regulating wound healing program. The increase in DNMT3a nuclear localization was time dependent, affecting wound proximal keratinocytes first and then propagates toward distal cells. At the completion of the wound healing program, we observe that DNMT3a localization is again prominent within the cytoplasms of differentiated (K5 negative) keratinocytes, while nuclear localization is restricted to cells in the basal layer of the epidermis (S2F Fig). In conclusion, we observe that the DNMT3a shows significant nuclear localization in the wound-proximal (leading edge) cells within 4 hours of the injury and the localization pattern further penetrates in the distal regions as time passes (Fig 2C). The nuclear localization kinetics also correlates with the pattern of caspase-8 down-regulation as well as promoter methylation (Fig 1B–1D).

### DNMT3a directly regulates caspase-8 expression

**Fig 3:**
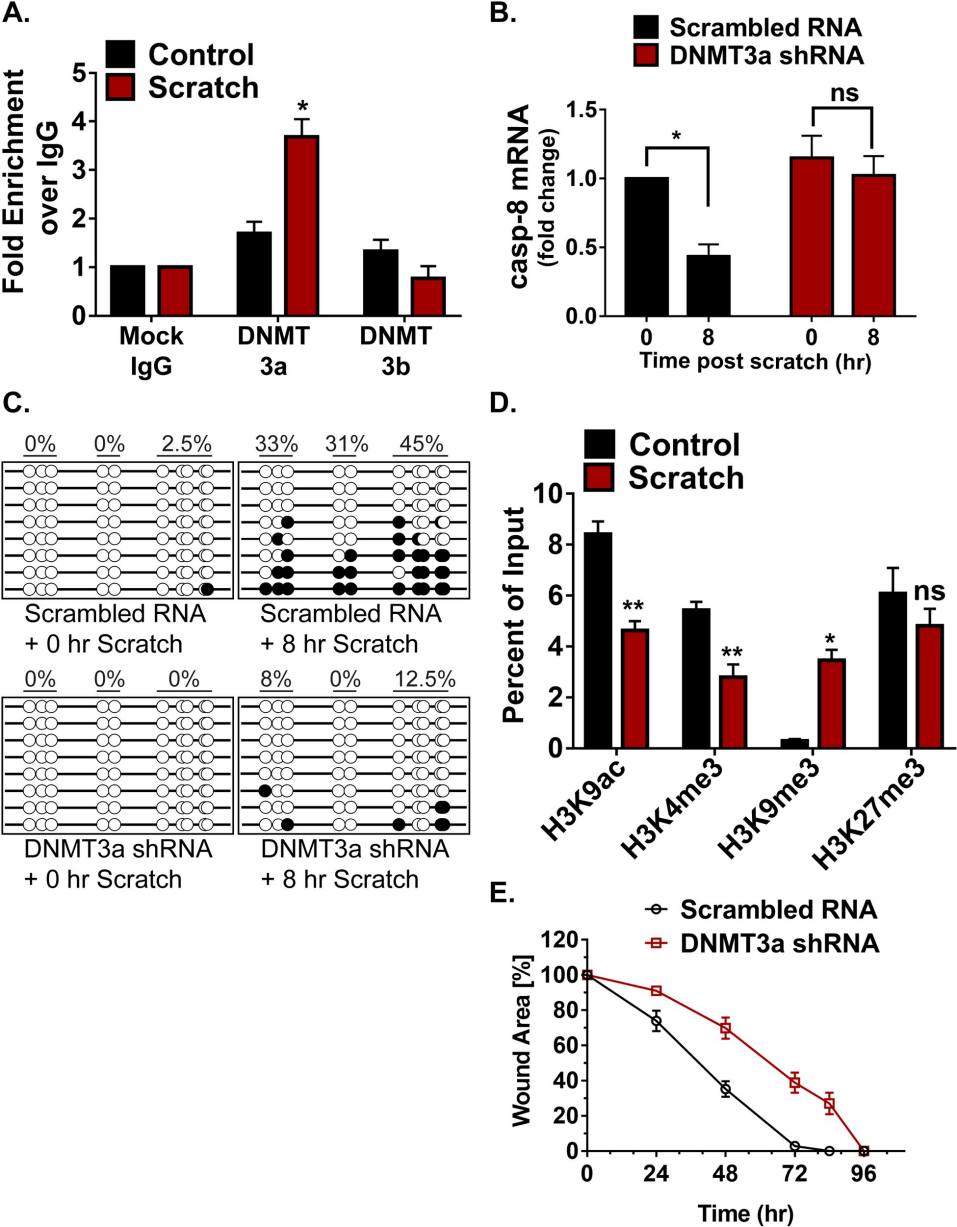
Involvement of DNMT3a and histone modification in regulating caspase-8 expression. (**A**) ChIP-qPCR analysis to check DNMT3a and DNMT3b occupancy at caspase-8 promoter in control and scratch wounded keratinocytes (*n* = 3). (**B**) qPCR analysis of caspase-8 mRNA in scratch wounded keratinocytes, transduced with either scrambled RNA or DNMT3a shRNA (*n* = 3). (**C**) DNA methylation status of caspase-8 promoter in scratch wounded keratinocytes, transduced with either scrambled RNA or DNMT3a shRNA. (**D**) ChIP-qPCR analysis of H3K9ac, H3K4me3, H3K9me3, and H3K27me3 at caspase-8 promoter in control and scratch wounded keratinocytes (*n* = 3). (**E**) Effect of DNMT3a down-regulation on in vitro wound healing assay (*n* = 3). (Data are shown as mean ± SEM, *P*-values were calculated using 2-tailed *t* test (A, B, D), * *P* ≤ 0.05, ** *P* ≤ 0.01, *** *P* ≤ 0.001, ns = *P* > 0.05.) Data underlying the graphs can be found in Fig 3A, 3B, 3D, and 3E of S1 Raw Data. ChIP, chromatin immunoprecipitation; DNMT3a, DNA methyltransferase 3A.

We further explored whether the de novo DNA methylation of caspase-8 promoter is the result of DNMT3a’s direct binding to this region (S1B Fig). This was accomplished with the use of chromatin immunoprecipitation (ChIP) to assess the level of DNMT3a occupancy on the caspase-8 promoter pre- and post-scratch wound. We found that scratch wounds lead to the higher occupancy of DNMT3a on caspase-8 promoter, which is not seen in the case of DNMT3b (Fig 3A). To understand the functional relevance of DNMT activity in maintaining caspase-8 levels, we pre-treated the differentiated keratinocytes with a generic DNMT inhibitor (5-Aza-2′-deoxycytidine). We observed that the inhibitor treated cells were unable to down-regulate caspase-8 mRNA in a scratch wound assay (S3A Fig). To specifically assess the role of DNMT3a, we performed shRNA-mediated knockdown of DNMT3a (S3B Fig). Compared to the controls, keratinocytes with reduced DNMT3a expression were unable to down-regulate caspase-8 in response to scratch wound (Fig 3B). We further analyzed whether failure of caspase-8 mRNA down-regulation was due to the absence of promoter methylation. Indeed, scratch wounded keratinocytes, transduced with DNMT3a shRNA, showed significantly reduced DNA methylation pattern on the caspase-8 promoter compared to scrambled RNA control (Fig 3C).

Promoter activities are often dependent on the associated histone modifications. These histone marks generally guide the DNA methylation at a particular genic region and vice-a-versa [21–23]. DNMT3a occupancy and activity has also been shown to be influenced by the methylation status of certain lysine (K) residues on the histone 3 (H3) tail [22,24]. To investigate the core machinery required for DNMT3a-mediated methylation on the caspase-8 promoter, we assessed several activation and repression histone marks in scratch wounded keratinocytes (Fig 3D). We observed that 2 transcriptional activation marks, H3K9ac and H3K4me3, are decreased at the caspase-8 promoter. On the other hand, the H3K9me3 mark, which is associated with transcriptional repression, was significantly increased at the caspase-8 promoter following wounding. Interestingly, another classical repressive mark, H3K27me3, did not show a significant change. It is possible that the caspase-8 proximal promoter is another example of a bivalent promoter [25] having both activation (H3K9ac and H3K4me3) and repression (H3K27me3) marks. In this scenario, then, wound-mediated repression of caspase-8 is achieved via reduction of both H3K9ac and H3K4me3 along with an increase in the H3K9me3 mark and DNMT3a occupancy. These results establish the mechanism by which DNMT3a localizes to the caspase-8 promoter. An outstanding question is whether DNMT3a is required for a proper wound healing response. To address this issue, we tested the effect of the knockdown of DNMT3a in a scratch wound assay (Fig 3E). We found that keratinocytes with decreased DNMT3a exhibited an impaired wound closure response, thereby illustrating the necessity of this methyltransferase in the proper repithelialization of an in vitro wound.

### Cellular tension mediates DNMT3a localization and caspase-8 expression

We observed that caspase-8 down-regulation and DNMT3a nuclear localization initiate at the edge of wound site (Figs 1 and 2). Given that these are early responses to injury, understanding the mechanistic basis of this phenomenon can provide insights into the broader process of cellular wound sensing. The keratinocytes in the epithelial sheet are strongly adhered to each other and an event of injury will result in the sudden relaxation in that tension, particularly in the cells at the boundary of the wound. Interestingly, the expanding number of cells exhibiting the down-regulation of caspase-8 RNA in the scratch wound assay over time (Fig 1A) closely parallels the changes in traction force previously reported for the collective cell migration of an epithelial sheet following a scratch wound [26]. We therefore investigated whether release of tension, caused by the severing of the epithelial sheet, can impact DNMT3a subcellular localization and subsequently caspase-8 expression. As shown in S4A Fig, modulation in cellular tension can be achieved via targeting the components of the adherens junction, which are known to play a role in generating and maintaining cellular tension [27,28].

**Fig 4:**
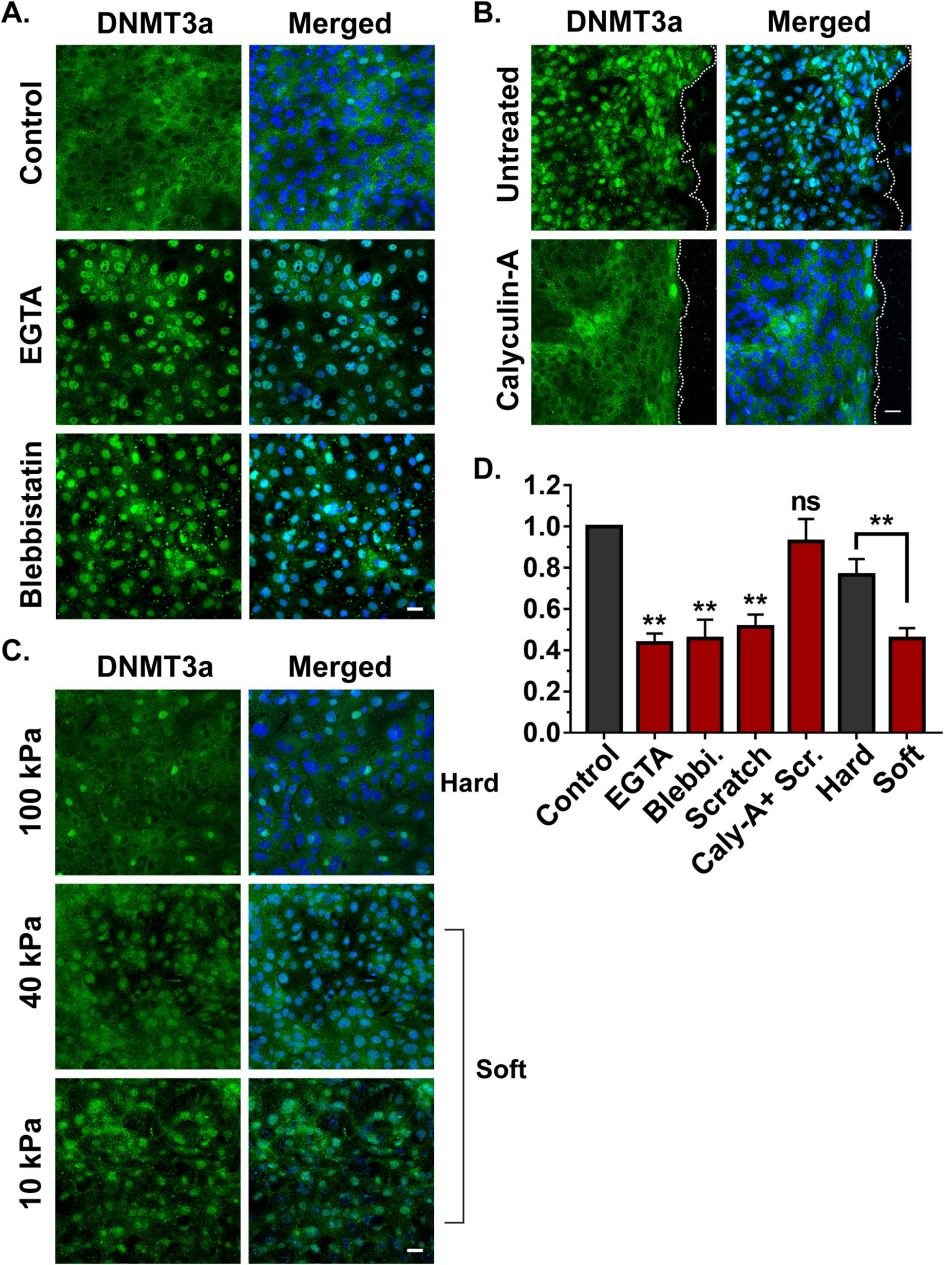
Effect of cellular tension on DNMT3a localization and caspase-8 expression. (**A**) Effect of EGTA and blebbistatin on the localization of DNMT3a. (**B**) Effect of scratch wound DNMT3a localization in presence and absence of calyculin-A. (**C**) Effect of various matrix stiffness on the localization of DNMT3a. (**D**) Fold change in the levels of caspase-8 mRNA as a result of varios pharmacological and mechanical approaches of tension modulation (*n* = 4), [scale bar = 20 μm]. (Data are shown as mean  ±  SEM, *P*-values were calculated using 2-tailed *t* test (D), * *P* ≤ 0.05, ** *P* ≤ 0.01, *** *P* ≤ 0.001, ns = *P* > 0.05). Data underlying the graphs can be found in Fig 4D of S1 Raw Data. DNMT3a, DNA methyltransferase 3A.

We observed that tension release by disrupting calcium-dependent E-cadherin junctions via EGTA treatment resulted in the nuclear localization of DNMT3a (Figs 4A and S4B). Similarly, releasing cellular tension endowed by nonmuscle myosin II (NM-II) with the pharmacological inhibitor of NMII, blebbistatin, induced the DNMT3a’s nuclear translocation from the cytosol (Figs 4A and S4B). Furthermore, we examined the effect of blocking release of cellular tension in a scratch wounded sheet of epidermal keratinocytes. The release of tension was blocked by pre-treating keratinocytes with calyculin-A, which inhibits myosin light-chain phosphatase, thereby maintaining the active state of NMII [29]. The treatment of keratinocytes with calyculin-A prior to scratch wounding blocked the nuclear translocation of DNMT3a that was observed in cells treated with vehicle control (Figs 4B and S4C).

In addition to a pharmacological approach, we also modulated cellular tension by altering the substrate stiffness on which the keratinocytes were growing. This was accomplished by utilizing polyacrylamide gels of various stiffness, which would alter cellular tension. We observed that differentiated keratinocytes seeded on “soft” matrices ranging from 10 kPa to 40 kPa mostly harbored DNMT3a in the nuclei (Figs 4C and S4D). However, cells grown on a “stiffer” matrix (100 kPa) predominantly showed a cytoplasmic localization of DNMT3a.

We then evaluated whether DNMT3a’s dynamic localization in response to pharmacological and mechanical alterations in cellular tension has any transcriptional consequences. We observed that in all the scenarios where DNMT3a nuclear localization was favored (scratch wounds, EGTA/blebbistatin treatment, soft substrates), caspase-8 RNA was down-regulated compared to their respective controls (Fig 4D). On the other hand, inhibition of DNMT3a’s nuclear localization (via calyculin-A or a stiff substrate) resulted in the failure of caspase-8 down-regulation in spite of a scratch wound. These results suggest a correlation between DNMT3a localization and changes in tensile forces. It should be noted that these interventions may have other effects on the cell in addition to modulating cellular tension, and thus we cannot rule out additional pathways leading to cellular reprograming via epigenetic means.

### DNA methylation could be a global regulator of gene expression to initiate wound healing program

We further assessed whether the down-regulation of caspase-8 is a paradigm for the global down-regulation of genes to achieve a cell state transition from homeostasis to wound healing. Surprisingly, the transcriptome profile of scratch wounded differentiated keratinocytes has not been reported even though these layers are the first to encounter damage in vivo. Thus, we performed RNA sequencing of wounded v/s unwounded primary mouse keratinocyte that were differentiated via the calcium switch protocol (S5A Fig). The analysis of the transcriptome data revealed that the number of down-regulated genes outnumbered the up-regulated genes post injury. We verified the sequencing data by specifically analyzing genes via qPCR that have already been implicated in wound healing or epidermal development (S5B Fig and S5C Fig). Interestingly, there was an inverse correlation with the RNA expression and the degree of methylation for many of the genes we interrogated. This suggest that DNA methylation could be a global regulator for a set of wound response genes (in addition to caspase-8) needed for the wound healing program.

Analysis of transcriptome data has revealed many such group of genes and their biological processes (S6 Fig). Of particular interest were the down-regulation of genes involved in the differentiation of keratinocytes. The Ghazizadeh lab has reported evidence of dedifferentiation of suprabasal keratinocytes as a mode of aiding cutaneous regeneration and repair. Interestingly, the regeneration of skin epithelia by differentiated epidermal cultures was found to be facilitated by the capacity of these cells to proliferate [30]. The transcriptome profile of scratch wounded differentiated keratinocytes reveals an up-regulation of cell cycle associated genes that is consistent with this report. Consequently, the convergence of mechanical and epigenetic cues appears to play an important role in the plasticity of differentiated epidermal keratinocytes in cutaneous repair and regeneration. The processes that occur during the wound healing phases of inflammation, proliferation, and tissue remodeling are often recapitulated in a deregulated manner in many pathologies leading to the notion of diseases with a “wound signature.” Prominent among these is the view of cancer as an over healing wound [31]. As we noted earlier, there is a body of literature demonstrating that the down-regulation of caspase-8 in cancer cells is accompanied with the methylation of its promoter region [32–35]. In addition, we have previously demonstrated that inflammatory human skin diseases such as atopic dermatitis [15] and psoriasis [16] likewise exhibit a loss of epidermal caspase-8. To probe a possible link between caspase-8 down-regulation and methyltransferase expression, we utilized the imiquimod-induced model of psoriasis in mice. In the psoriatic skin of mice, we observed robust nuclear localization of DNMT3a in all the epidermal layers, whereas in the control animals, nuclear DNMT3a was primarily localized in the basal keratinocytes (S5D Fig). Altogether, this suggests that the epigenetic regulation governing the cell state transition in wound healing is usurped in many diseases ranging from inflammatory skin diseases to carcinomas.

## Discussion

The wound healing literature involving epidermal keratinocytes have elegantly described many signaling pathways and gene expression profiles in the proliferating cells of the basal layer [36,37]. In contrast, differentiated epidermal cells, such as the suprabasal keratinocytes near the outer surface of the skin, have largely been overlooked for their potential role during wound healing. Interestingly, our previous work demonstrates that the uppermost layer of differentiated keratinocytes, namely the granular layer, expresses caspase-8 that has a non-canonical role in regulating the wound healing program [12]. It was found that the down-regulation of caspase-8 is both necessary and sufficient to induce a wound healing response in the absence of any tissue damage. In addition, the chronic down-regulation of caspase-8 underlies inflammatory skin diseases such as atopic dermatitis [15] and psoriasis [16]. These findings have made the decrease in caspase-8 expression a useful wound healing biomarker and led us to inquire about the mechanism of caspase-8 regulation in skin keratinocytes.

Clues about the regulation of caspase-8 are reported in the context of cancer. Similar to the wound healing process, it is generally down-regulated in various cancers [18,38,39]. It is possible that the cancers, known as “over healing wound,” usurp physiological pathway of wound healing for its own propagation [31]. Here, we show that wound sensing leads to the acute increase in the caspase-8 promoter methylation as a potential mechanism of gene silencing. This parallels with the findings on caspase-8 down-regulation in hepatocellular carcinoma, where methylation status of SP1 sites and nearby CpG dinucleotides in the promoter region were proposed to be a major regulator of caspase-8 expression [19]. In fact, it has been observed that caspase-8 and several other genes are known to be down-regulated in various cancers via DNA methyltransferase (DNMT) activity [18,38,39]. The overexpression of DNMT3a has also been shown to be associated with several cancers [40,41]. The process of de novo DNA methylation during an acute physiological response such as wound healing is a rarely described phenomenon. Mammalian cells are known to have only 2 de novo DNA methyltransferases, DNMT3a and DNMT3b. Both have been widely studied for their role in physiological processes like embryogenesis [42] and hematopoiesis [43], as well as pathological conditions such as cancer [44,45]. In particular, it has been shown that DNMT3a and 3b are required as regulators of enhancer activity and RNA production of genes necessary for epidermal stem cell homeostasis [4]. In disease context, DNMT3a has been described to be overexpressed or mutated in various carcinomas [46,47] and correlates with the down-regulation of caspase-8 in these same scenarios. Here, we found that upon injury to the skin or differentiated epidermal sheets, the suprabasal cells near wound edge showed a nuclear localization of DNMT3a, but not DNMT3b. We have captured that DNMT3a indeed occupies the caspase-8 promoter and plays an important role in its down-regulation post injury. Importantly, there are numerous additional promoters that also methylated and gene expression is transcriptionally down-regulated. This indicates that DNA methyltransferases have a broad spectrum of genomic targets that work in combination to fuel the cell state transition from homeostasis to repair. In parallel to the DNA methylation, the literature also describes changes in histone modifications responsible for the ON/OFF state of a particular gene. The histone modifications and their modifiers have been studied in depth to understand how the expressions of various epidermal differentiation genes are regulated [48]. In general, H3K9ac and H3K4me3 are considered as gene activation marks and H3K9me3 and H3K27me3 are known as repression marks. It is also observed that certain methylation state of H3K36 dictates the DNMT3a’s recruitment to a particular DNA segment on the chromosome [49,50]. In our efforts to understand the histone modifications during wound healing, we observed a reduction in H3K9ac and H3K4me3 levels, along with an increase in the H3K9me3 mark at the caspase-8 promoter. These histone modifications are known to be regulated via various other epigenetic players such as polycomb repressive complexes (PRC 1/2), JMJD, Setd8, and HDACs during epidermal development [48].

How these epigenetic players are regulated is another important question in the field. While there are many chemical cues, adhesion signals, and transcription factors described to regulate the wound healing process, emerging evidence links mechanical forces to epigenetic and transcriptional responses [51,52]. Even during the development of epidermal tissue, tension generating molecular players like nonmuscle myosin IIA (NMIIA), along with emerin (Emd) and PRC2 regulate the differentiation process of epidermal stem cells. The strain on epidermal cells reduces Emd levels from the inner nuclear membrane, which then leads to the loss of the histone mark H3K9me2,3. This is followed by PRC2 mediated increase of H3K27me3 occupancy at several heterochromatic regions and thereby gene silencing [53]. Along the same line, recently Nava and colleagues has described how short- and long-term mechanical stress on a cell can result in changes in stiffness of the nuclear membrane, loss of H3K9me3 marks at the heterochromatin, and overall chromatin and cytoskeletal reorganization [54]. These are some of the key discoveries suggesting external mechanical forces drive changes in heterochromatin organization, gene expression changes, and cytoskeletal reorganization in a way that mechanical energy gets redistributed and DNA damage can be avoided. In this context, our results demonstrate that the release in the mechanical tension, either by physical or chemical treatments, results in the DNMT3a’s nuclear localization and down-regulation of caspase-8. This observation is consistent with the concept of mechano-sensitive histone modifications, which could lay a foundation for the occupancy of DNMT3a. In a wider context of cellular reprogramming during the wound response, mechanotransduction seem to have a large impact on the transcriptome of the cell via the concomitant initiation of several epigenetic pathways. Future studies in this area will include elucidation of the connection between the release of mechanical tension and their sensing by these epigenetic machineries. For example, DNMT3a has been shown to have multiple binding partners (DNMT3L, SUMO-1, Cbx4, Ubc9, RP58, HDAC1) for their nuclear shuttling as well as chromosomal occupancy, some of which can potentially function as a primary signal sensor to guide the localization of DNMT3a [55,56]. Moreover, in different cell types, changes in mechanical tension have been documented to directly induce the nuclear translocation of important transcription factors. A notable example of which is the YAP/TAZ complex, which has proliferation stimulating gene targets [57].

The described model of mechanosensitive epigenetic players would obviously be regulating a larger gene regulatory network, in addition to caspase-8. Interestingly, the transcriptome literature on wound healing has utilized proliferating keratinocytes, leaving the transcriptome profile of differentiated keratinocytes unknown despite the fact that it constitutes about 2/3 of the epidermis. Our research fills an important gap by providing a transcriptome profile of in vitro wounded differentiated keratinocytes. The results give us a unique insight in the regulation of various unexplored wound-response genes. On a particular note, we observe a strong down-regulation of multiple epidermal differentiation genes in response to injury. From the current transcriptome and literature survey, it is evident that various keratinocyte differentiation markers (such as involucrin, keratins K1/K10, and filaggrin) are down-regulated along with cell adhesion molecules (involved in tight junction, adherens junctions, and desmosomes). This is consistent with a report from S. Ghazizadeh’s lab that de-differentiation of suprabasal keratinocytes is a contributing factor in the wound healing response [30]. Our data suggest that the release of mechanical tension in differentiated keratinocytes is one component in this process by inducing a “partial de-differentiation” and perhaps additional soluble signaling cues are required to achieve complete dedifferentiation.

## Materials and method

### Ethics statement

All animal work was approved by the Institutional Animal Ethics Committee in the CJ lab (INS-IAE-2019/06[R1]). Experiments on mice followed the norms specified by the Committee for the Purpose of Control and Supervision of Experiments on Animals (Government of India). All experimental work was approved by the Institutional Biosafety Committee of inStem (inStem/G-141(3)/2012 and inStem/G-141(3)-06/2016).

### Cell culture and scratch wound assay

The isolation of primary keratinocytes from neonatal mice was performed as described in [58]. Briefly, mice pups were sacrificed and the skin was removed. The skin was kept in dispase at 4°C overnight (or 37°C for 1 hour) to separate epidermis. The epidermis was then digested with trypsin to isolate keratinocytes. These cells were filter with 70-μm mesh and cultured further as described in (Nowak and colleagues, 2009) [59]. The keratinocytes were cultured in lab with feeder cells (3T3J2) for 10 passages. Then, feeder-independent keratinocytes were taken and tested for their differentiation potential via calcium switch protocol [60]. Various differentiation markers were checked via qPCR. The batch of cells showing proper differentiation and morphology were then selected for further experiments.

Proliferating keratinocytes were maintained in low Ca^2+^ E-media (0.05 mM). For differentiation, they were allowed to reach 100% confluence and then introduced with high Ca^2+^ (1.2 mM) E-media for 48 hours. Once they differentiated and appear as sheet-like morphology, scratch wounds were made (with the help of a 1-ml tip) at multiple sites in each culture plate. To keep the constancy between experiments, the distance between the consecutive scratch was kept approximately 0.5 mm. The scratch wounds were followed by a 1× PBS wash, and fresh high Ca^2+^ (1.2 mM) E-media were added to each plate. As described in the figure legends, the cells were harvested at several time points using TRIzol reagent for RNA isolation or using lysis buffer for DNA isolation.

### Mice

C57Bl6/J animals were originally purchased from Jackson Laboratory (Stock No. 000664) and were bred for >10 generations in the NCBS vivarium facility. The 8-week-old mice were anesthetized, and 5-mm or 8-mm punch biopsies were used to make full-thickness excisional wounds.

### Tissue section and staining

Wounded regions were embedded in OCT, frozen on dry ice, and stored in −80^o^ freezer for further sectioning and antibody staining, and 10- to 15-μm section were taken, stained with primary antibody at 4°C overnight, and then with secondary antibody at RT for 20 to 30 minutes. Antibodies used in this study are as following: Caspase-8 (Enzo #ALX-804-447-C100), DNMT3a (Abcam #ab2850, SC #365769), K5 (lab generated). Sections were imaged using IX73 Olympus microscope.

### In situ hybridization

DIG labeled 5′ mouse caspase-8 cRNA probe was synthesized as per the manufacturer’s instructions (Roche dig labeling kit–# 11175025910). In situ hybridization was performed as described earlier [61]. Briefly, the paraffin tissue sections were deparaffinized by treatment by xylene and ethanol gradient, or the 4% PFA fixed cells were permeabilized using 0.2% TritonX-100 for 10 minutes at room temperature, and 5 ng DIG labeled cRNA probes per 100 μL hybridization buffer was applied on the sections overnight at 63°C. Same concentration of DIG labeled mRNA with the complimentary sequence to cRNA was used as a negative control. Washing was done at 65°C. The Anti-DIG antibody (Roche # 11093274910 Roche) was applied overnight as per manufacturer’s instructions. Sections were developed for 30 minutes at 37°C using BCIP/NBT solution (Sigma # B6404). Reaction was stopped using de-ionized water once the purple color was developed. Sections were mounted using MOWIOL solution and imaged using bright-field microscope.

### Hydrogel of varying stiffness

The polyacrylamide-based hydrogels were prepared as describe in [62,63]. They were coated with collagen and seeded with enough cells to make it 80% to 100% confluent and were allowed to settle for 24 to 48 hours before initiating the keratinocyte differentiation.

### Chromatin immunoprecipitation (ChIP)

ChIP of histone modification was performed as described previously [64] with some modifications. In brief, harvested keratinocytes (unscratched and scratched) were cross-linked with 1% formaldehyde. Cells were lysed in buffer N containing DTT, PMSF, and 0.3% NP-40. After isolation of nuclei, chromatin fractionation was done using 0.4 U of MNase (N5386, Sigma) at 37°C for 10 minutes. Reaction was stopped using MNase stop buffer without proteinase K. Simultaneously, antibodies against H3K27me3, H3K9me3, H3K4me3, H3K9ac, and Rabbit IgG were kept for binding with Dynabeads for 2 hours at RT. After equilibration of beads, chromatin was added for pre-clearing. To antibody bound beads, pre-cleared chromatin was added and kept for IP at 4°C overnight. Next day, beads were washed and eluted at 65°C for 5 minutes. Eluted product was subjected to reverse cross-linking along with input samples, first with RNase A at 65°C overnight and then with Proteinase K at 42°C for 2 hours. After reverse cross-linking, DNA purification was performed using phenol:chloroform extraction method. Antibodies used for this protocol are listed here: H3K27me3 (07–449, Milipore), H3K9me3 (ab8898, Abcam), H3K9ac (ab4441, Abcam), H3K4me3 (ab8580, Abcam).

### Bisulphite reaction, sequencing, and analysis

Genomic DNA was isolated by salting out method as described elsewhere [65], then treated with RNase for 1 hour at 37°C. Further, approximately 20 μg DNA was taken in 200 μL volume and purified with phenol:chloroform extraction method. The purified DNA was checked for its integrity via running on the agarose gel. The DNA sample having good integrity and free of RNA were taken for bisulphite conversion as per manufacturer’s protocol (Zymo #D5005). The converted DNA was then amplified using bisulphite conversion-specific primers, the amplified product was assessed on the agarose gel, ligated with TOPO-TA vector, and transformed in competent Top10 cells. Two to 3 colonies from each experiment were sent for Sanger’s sequencing using caspase-8 promoter-specific sequencing primers (GAATAAGGAAGTGTTTTTTAG, AAAACTATACTCACTTCCTATTC). The sequenced file (FASTA) was uploaded to http://quma.cdb.riken.jp/ for CpG methylation analysis.

### Lentivirus shRNA constructs and transduction

Plasmids expressing shRNAs were obtained from TransOmics (DNMT3a # TLMSU1400). To produce viruses, HEK293T cells were transfected with psPAX2, pMD2.G, and either non-targeting random RNA sequence vector or shRNA-containing plasmids, using Lipofectamine transfection reagent according to the manufacturer’s protocol. Following a 48- to 72-hour transfection, the virus particle-containing media was collected, concentrated with filters, and added to the differentiated cells for 24 hours. Expression of DNMT3a was measured 2 to 3 days after viral infection. Silencing efficiency was confirmed by immunoblotting.

### Quantitative real-time PCR

RNA was isolated from human keratinocytes (proliferating or differentiated) using the RNAiso Plus (Takara), and 1 μg of RNA was used to prepare cDNA using the PrimeScript kit (Takara). cDNA equivalent to 100 ng of RNA was used for setting up the qPCR reaction using the SYBR green 2× master mix. All reactions were performed in technical triplicates using the CFX384 Touch Real-Time PCR detection system (BioRad). Primers used in this study are listed here: caspase-8 mRNA (TCTGCTGGGAATGGCTACGGTGAA, GTGTGAAGGTGGGCTGTGGCATCT), caspase-8 promoter (GGGAATAAGGAAGTGTCCTCCA, CCCAGAACTGTACTCACTTCCTG), beta actin (GGGCTATGCTCTCCCTCAC, GATGTCACGCACGATTTCC).

### RNA sequencing and data analysis

The scratch wounded cells and controls were collected after 8 hours in TRIzol reagent and RNA was isolated using standard TRIzol-based RNA isolation method. The library preparation and NGS RNA sequencing steps were outsourced to a commercial facility (Genotypic). Once the raw sequencing reads were received, sequencing data analysis was performed using the following analysis pipeline. Briefly, raw sequencing data was QC checked with the “FASTQC” tool (Babraham Bioinformatics). Adapter contamination and bad quality reads were trimmed using “Trimmomatic” tool [66]. The good quality reads were then mapped to mm10 (mouse) reference genome using “HISAT2” [67]. The resulting “SAM” outputs were converted to “BAM” output and sorted. The “HTSeq-Count” tool was used to generate expression matrix from all 4 samples. Then, differential expression was analyzed with the help of DESeq2 R package.

### Gene ontology enrichment analysis

To explore enrichment of Gene Ontology among the significantly down-regulated (*n* = 428) and up-regulated genes (*n* = 358), we have used http://geneontology.org/ resources that runs “PANTHER” for the enrichment analysis [68].

Additional details of the NGS RNA seq samples are given in Table 1.

**Table 1:** NGS read counts.

Sample names	Raw sequencing read counts	Good quality read counts (used for mapping to mm10 reference genome)
Control-1	33693828	30441624
Control-2	34605485	30973898
Scratch wound-1	32255293	27735961
Scratch wound-2	31056628	27761854

## Supporting information

S1 Fig Caspase-8 RNA half-life and CpG positions on its promoter proximal region. (**A**) Quantification of caspase-8 mRNA to check its half-life post transcriptional block (using Actinomycin-D) (*n* = 3). (**B**) In situ hybridization with anti-sense and sense probe of caspase-8 RNA (in vitro) [scale = 10 μm]. (**C**) In situ hybridization with anti-sense and sense probe of caspase-8 RNA (in vivo) [scale = 20 μm]. (**D**) Model showing positions of CpG dinucleotide and SP1 binding sites in caspase-8 promoter proximal region. (Data are shown as mean ± SEM, *P*-values were calculated using 1-way ANOVA with Dunnett’s test (A), * *P* ≤ 0.05, ** *P* ≤ 0.01, *** *P* ≤ 0.001, ns = *P* > 0.05.) Data underlying the graphs can be found in S1A Fig of S1 Raw Data.
(TIF)

S2 Fig (**A**) Representative image of unwounded/wound-distal skin section stained with DNMT3a, DAPI, and K5. (**B’**) A model showing the quantification method of DAPI and DNMT3a stain intensities over the line of interest (1, 2) from proliferating and differentiated keratinocytes, followed by (**B”**) the plots of intensity values (gray unit) (calculated intensities from 4 biological replicates). Staining of in vitro proliferating and differentiated keratinocytes with (**C**), DNMT3a/DAPI and (**D**), DNMT3b/DAPI. (**E**) DNMT3b/DAPI staining of scratch wounded in vitro differentiated keratinocytes. (**F**) DNMT3a western blot analysis from control and scratch wounded keratinocytes at 8-hour time point (**G**), DNMT3a, DAPI, and K5 staining of a completely healed mouse skin section [scale = 20 μm]. Data underlying the graphs can be found in S2B Fig of S1 Raw Data.
(TIF)

S3 Fig (**A**) qPCR analysis of caspase-8 mRNA in scratch wounded keratinocytes, pre-treated with 5-Aza-2′-deoxycytidine (5A-dC) or DMSO (*n* = 4) (**B**), western blot analysis from keratinocytes transduced with scrambled RNA or DNMT3a shRNA (α-Tub = alpha-tubulin) (data are shown as mean ± SEM, *P*-values were calculated using 2-tailed *t* test (A), * *P* ≤ 0.05, ** *P* ≤ 0.01, *** *P* ≤ 0.001, ns = *P* > 0.05). Data underlying the graphs can be found in S3A Fig and S3B Fig of S1 Raw Data.
(TIF)

S4 Fig (**A**) Model showing various potential protein molecules (red labels) involved in generating and/or sensing the cellular tension. (**B**) Quantification of DNMT3a localization (nuclear v/s cytoplasmic) in EGTA and blebbistatin-treated keratinocytes compared to control (*n* = 3). (**C**) Quantification of DNMT3a localization in scratch wound proximal (≤100 μm) keratinocytes comparing control and calyculin-A-treated scratch wounds (*n* = 3). (**D**) Quantification of DNMT3a localization in (*n* = 3). (Data are shown as mean ± SEM, *P*-values were calculated using 2-tailed *t* test (D), * *P* ≤ 0.05, ** *P* ≤ 0.01, *** *P* ≤ 0.001, ns = *P* > 0.05.) Data underlying the graphs can be found in S4B–S4D Fig of S1 Raw Data.
(TIF)

S5 Fig Methylation-induced transcriptional reprogramming of epidermal keratinocytes from homeostasis to repair. (**A**) Heat map of differentially regulated genes in control and scratch wounded keratinocytes. (**B**) Scratch wound induced transcriptional down-regulation of genes and status of their associated DNA methylation levels. (**C**) Fold change of transcriptionally up-regulated genes and their associated DNA methylation levels (MeDIP-qPCR, y-axis = fold change compared to control). (**D**) DNMT3a and caspase-8 staining of control and psoriatic mouse skin (induced through imiquimod treatment), [scale bar = 100 μm]. Data underlying the graphs can be found in S5A–S5C Fig of S1 Raw Data. DNMT3a, DNA methyltransferase 3A; MeDIP, Methylated DNA Immunoprecipitation.
(TIF)

S6 Fig Gene ontology of up-regulated and down-regulated genes (Biological Processes). Processes are listed as–Log_10_ of adjusted FDR values. Top 15 relevant biological processes are chosen for generating the graphs. Data underlying the graphs can be found in S6 Fig of S1 Raw Data.
(TIF)

S1 Raw Data Raw data for Figs 1–4 and S1–S6. (ZIP)

## Abbreviations:

ChIP: chromatin immunoprecipitation
DNMT: DNA methyltransferase
DNMT3a: DNA methyltransferase 3A
MeDIP: Methylated DNA Immunoprecipitation
miRNA: micro-RNA
NM-II: nonmuscle myosin II
PRC2: polycomb repressive complex 2
