# Supplementary figures and images for: Initiation of wound healing is regulated by the convergence of mechanical and epigenetic cues
Source: PLoS Biol. 2022 Sep 16;20(9):e3001777. doi: 10.1371/journal.pbio.3001777 (PMC9522318; doi:10.1371/journal.pbio.3001777)

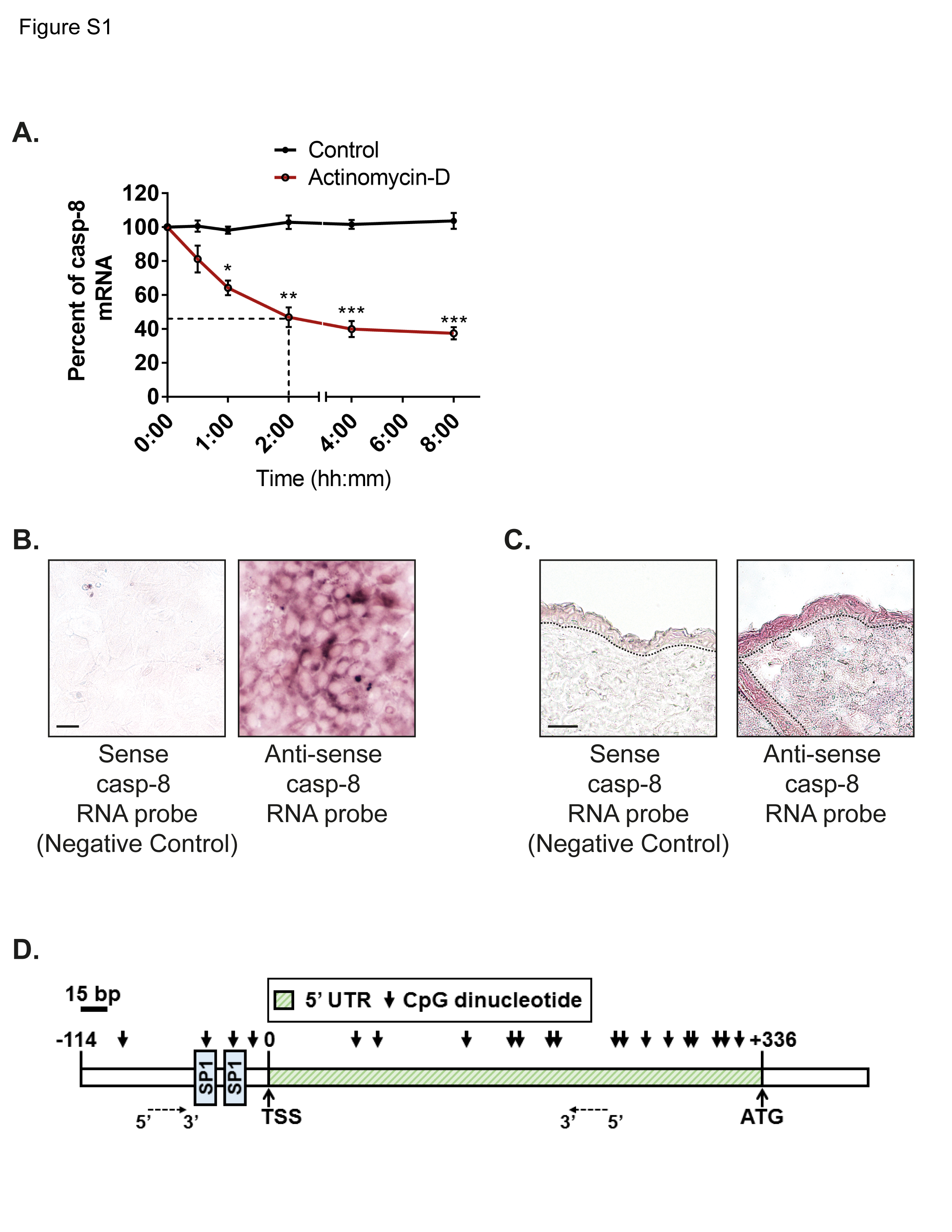

Supplement: S1 Fig — (A) Quantification of caspase-8 mRNA to check its half-life post transcriptional block (using Actinomycin-D) (n = 3). (B) In situ hybridization with anti-sense and sense probe of caspase-8 RNA (in vitro) [scale = 10 μm]. (C) In situ hybridization with anti-sense and sense probe of caspase-8 RNA (in vivo) [scale = 20 μm]. (D) Model showing positions of CpG dinucleotide and SP1 binding sites in caspase-8 promoter proximal region. (Data are shown as mean ± SEM, P-values were calculated using 1-way ANOVA with Dunnett’s test (A), * P ≤ 0.05, ** P ≤ 0.01, *** P ≤ 0.001, ns = P > 0.05.) Data underlying the graphs can be found in S1A Fig of S1 Raw Data. (TIF) [file pbio.3001777.s001.tif]

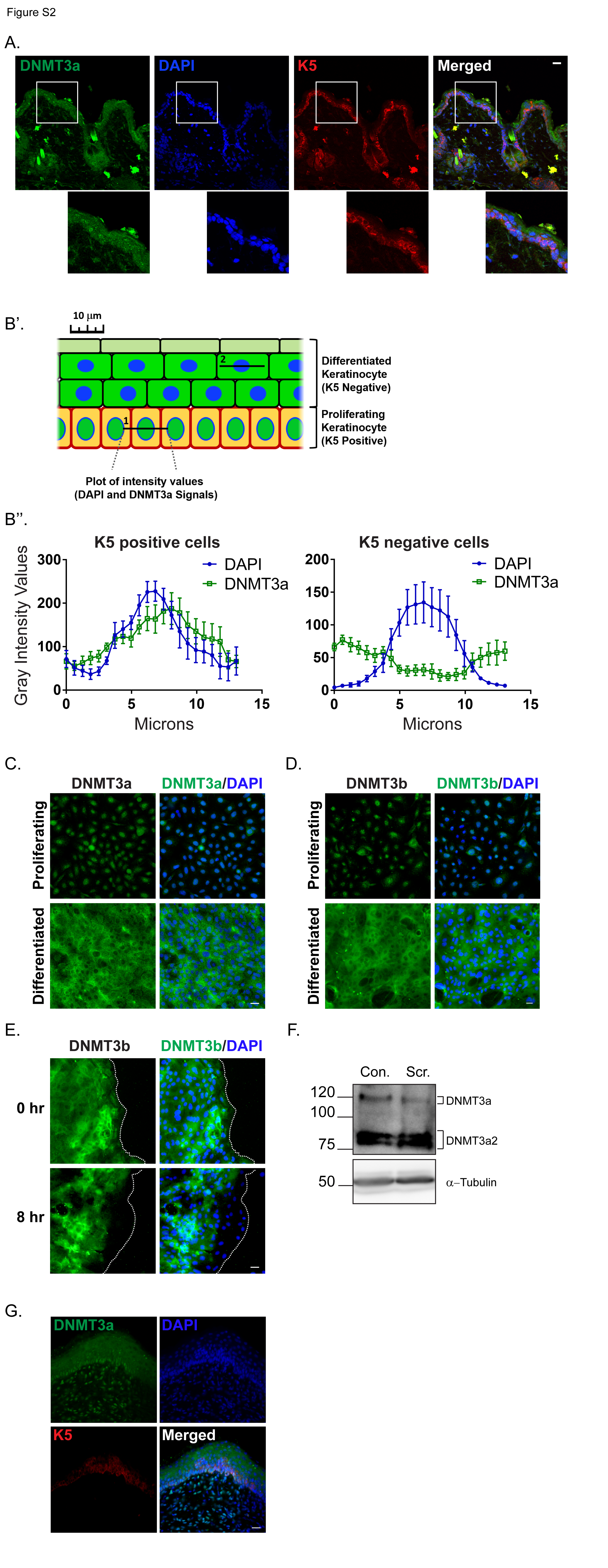

Supplement: S2 Fig — (A) Representative image of unwounded/wound-distal skin section stained with DNMT3a, DAPI, and K5. (B’) A model showing the quantification method of DAPI and DNMT3a stain intensities over the line of interest (1, 2) from proliferating and differentiated keratinocytes, followed by (B”) the plots of intensity values (gray unit) (calculated intensities from 4 biological replicates). Staining of in vitro proliferating and differentiated keratinocytes with (C), DNMT3a/DAPI and (D), DNMT3b/DAPI. (E) DNMT3b/DAPI staining of scratch wounded in vitro differentiated keratinocytes. (F) DNMT3a western blot analysis from control and scratch wounded keratinocytes at 8-hour time point (G), DNMT3a, DAPI, and K5 staining of a completely healed mouse skin section [scale = 20 μm]. Data underlying the graphs can be found in S2B Fig of S1 Raw Data. (TIF) [file pbio.3001777.s002.tif]

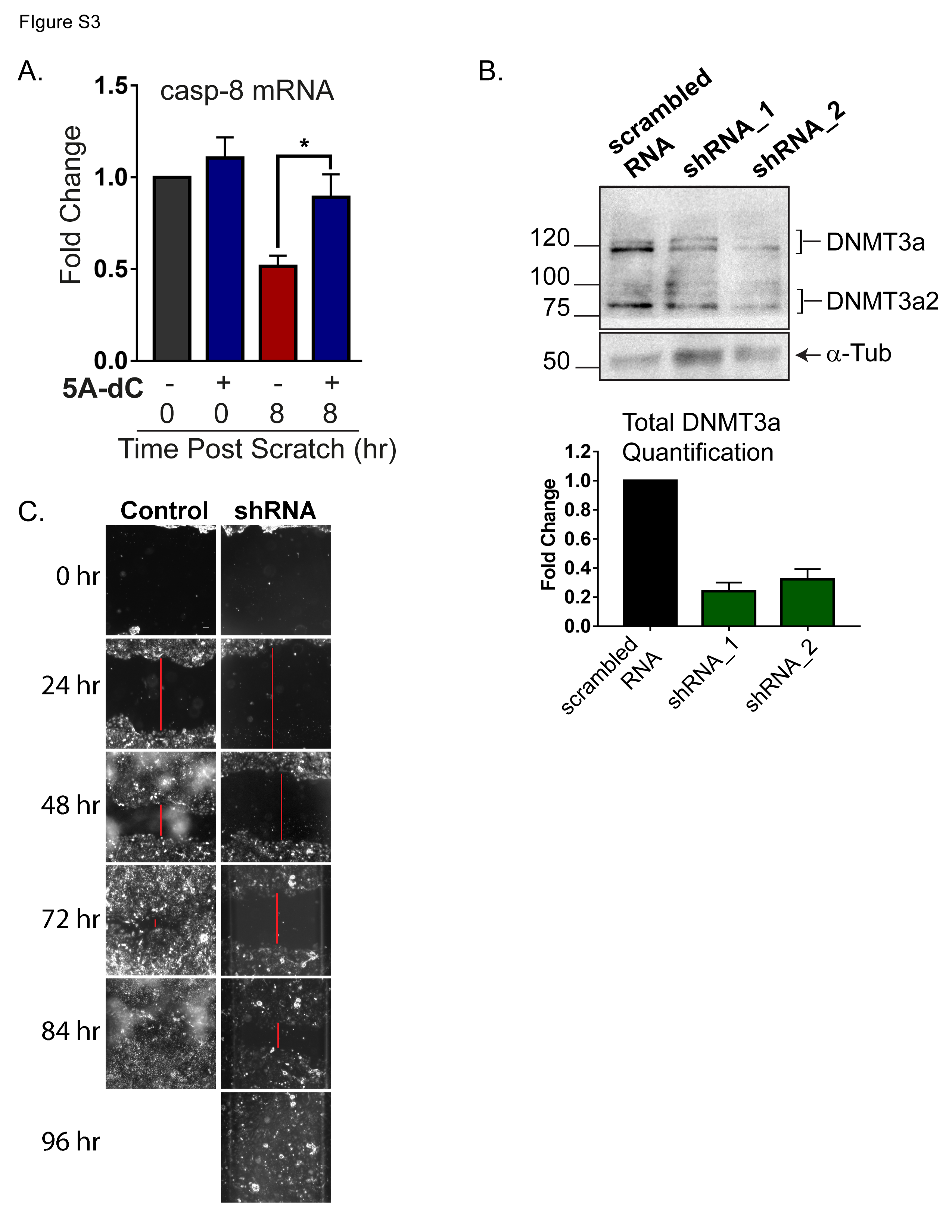

Supplement: S3 Fig — (A) qPCR analysis of caspase-8 mRNA in scratch wounded keratinocytes, pre-treated with 5-Aza-2′-deoxycytidine (5A-dC) or DMSO (n = 4) (B), western blot analysis from keratinocytes transduced with scrambled RNA or DNMT3a shRNA (α-Tub = alpha-tubulin) (data are shown as mean ± SEM, P-values were calculated using 2-tailed t test (A), * P ≤ 0.05, ** P ≤ 0.01, *** P ≤ 0.001, ns = P > 0.05). Data underlying the graphs can be found in S3A Fig and S3B Fig of S1 Raw Data. (TIF) [file pbio.3001777.s003.tif]

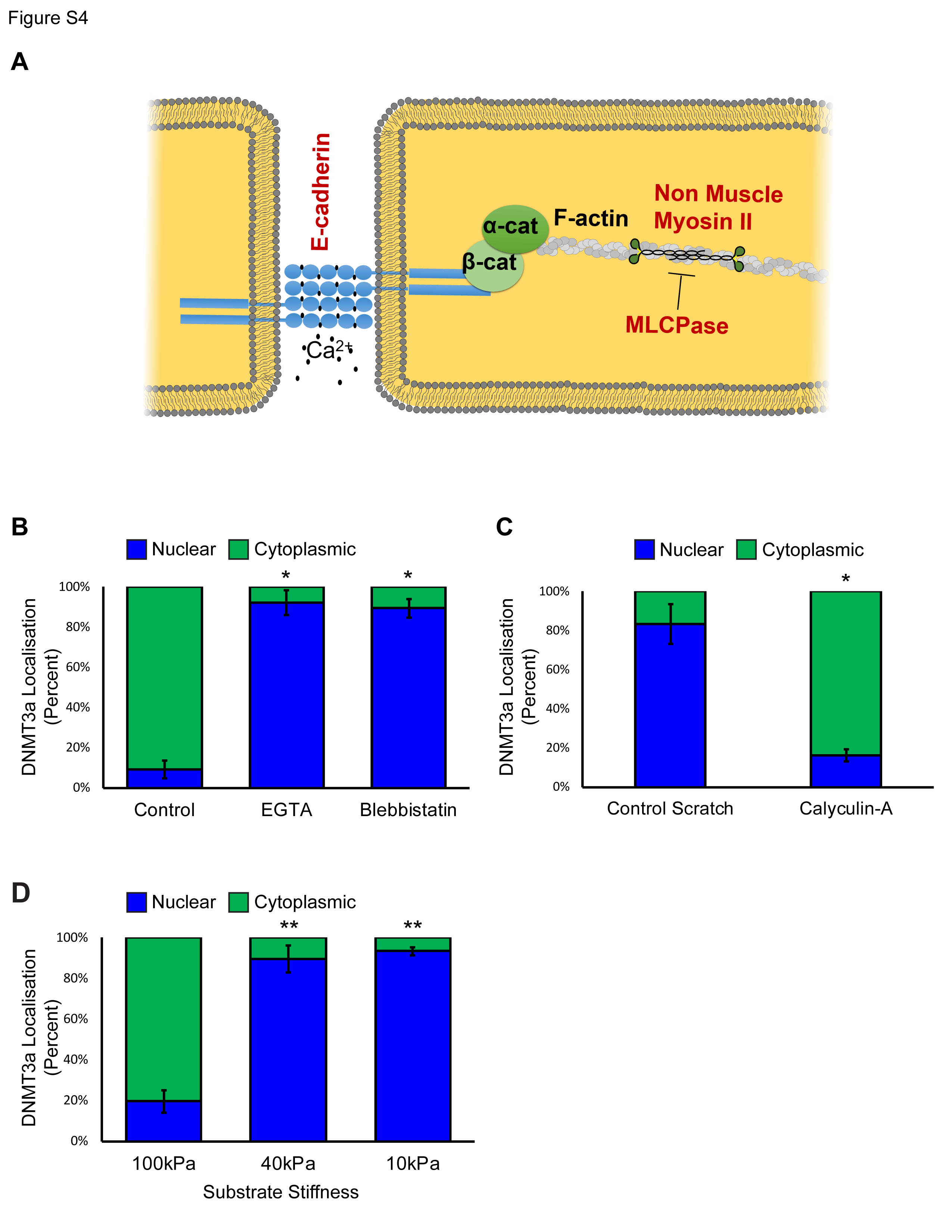

Supplement: S4 Fig — (A) Model showing various potential protein molecules (red labels) involved in generating and/or sensing the cellular tension. (B) Quantification of DNMT3a localization (nuclear v/s cytoplasmic) in EGTA and blebbistatin-treated keratinocytes compared to control (n = 3). (C) Quantification of DNMT3a localization in scratch wound proximal (≤100 μm) keratinocytes comparing control and calyculin-A-treated scratch wounds (n = 3). (D) Quantification of DNMT3a localization in (n = 3). (Data are shown as mean ± SEM, P-values were calculated using 2-tailed t test (D), * P ≤ 0.05, ** P ≤ 0.01, *** P ≤ 0.001, ns = P > 0.05.) Data underlying the graphs can be found in S4B–S4D Fig of S1 Raw Data. (TIF) [file pbio.3001777.s004.tif]

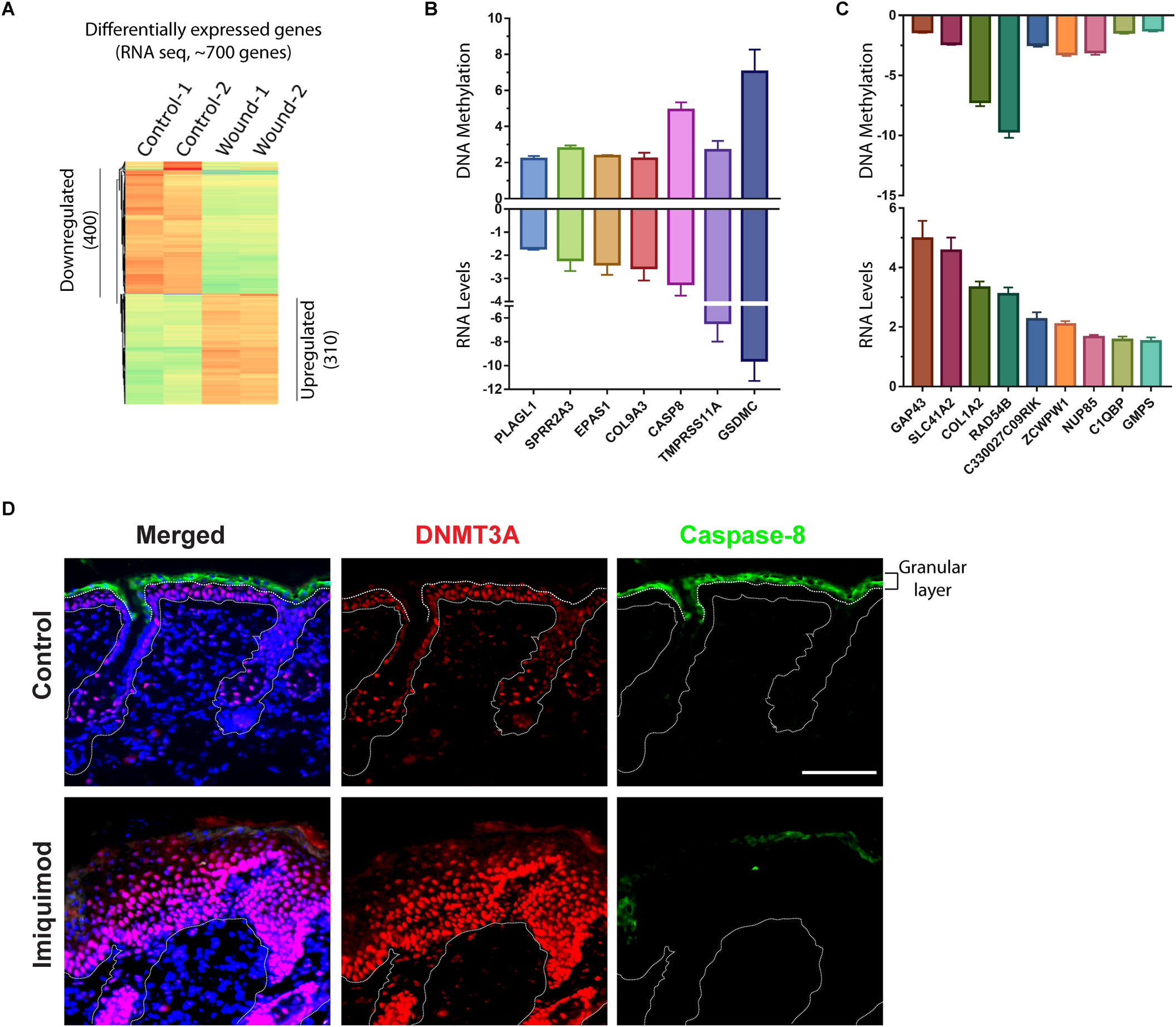

Supplement: S5 Fig — (A) Heat map of differentially regulated genes in control and scratch wounded keratinocytes. (B) Scratch wound induced transcriptional down-regulation of genes and status of their associated DNA methylation levels. (C) Fold change of transcriptionally up-regulated genes and their associated DNA methylation levels (MeDIP-qPCR, y-axis = fold change compared to control). (D) DNMT3a and caspase-8 staining of control and psoriatic mouse skin (induced through imiquimod treatment), [scale bar = 100 μm]. Data underlying the graphs can be found in S5A–S5C Fig of S1 Raw Data. DNMT3a, DNA methyltransferase 3A; MeDIP, Methylated DNA Immunoprecipitation. (TIF) [file pbio.3001777.s005.tif]

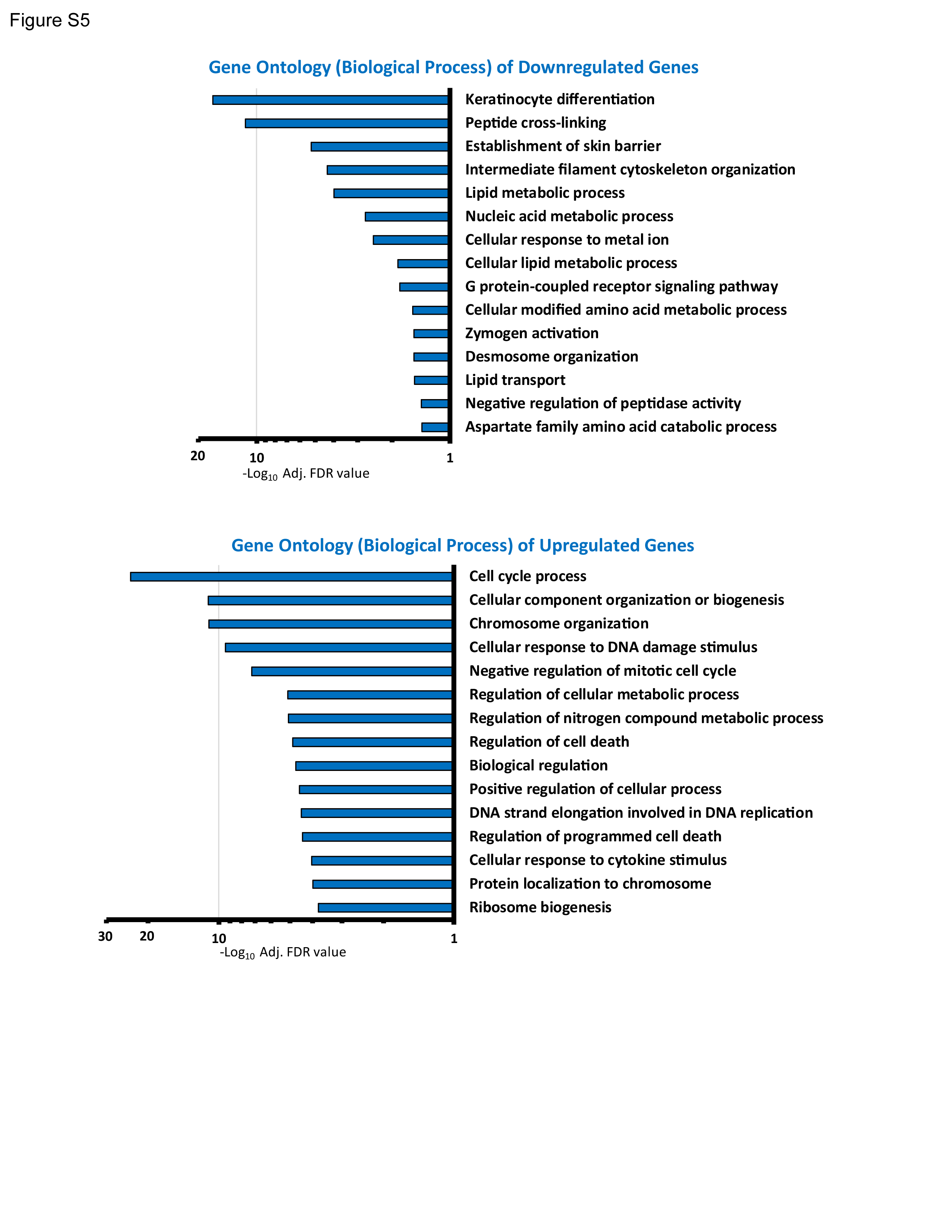

Supplement: S6 Fig — Processes are listed as–Log10 of adjusted FDR values. Top 15 relevant biological processes are chosen for generating the graphs. Data underlying the graphs can be found in S6 Fig of S1 Raw Data. (TIF) [file pbio.3001777.s006.tif]

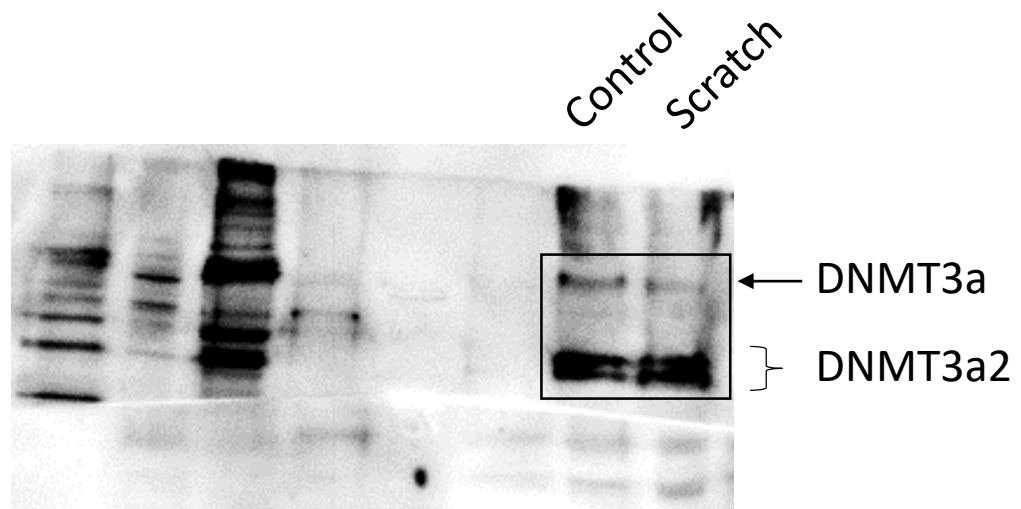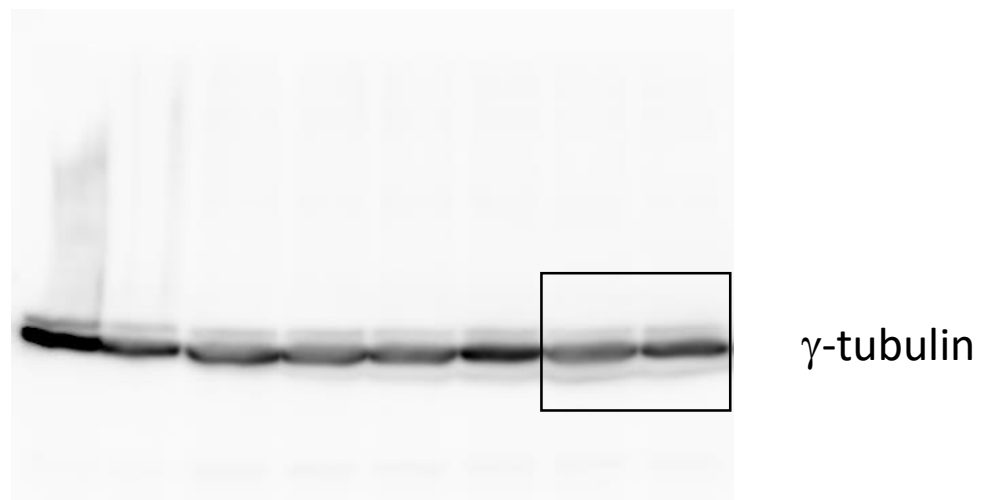

Supplement: S1 Raw Data — (ZIP) [file pbio.3001777.s007.zip › Supporting Information Raw Data/S2F_Fig.pdf]

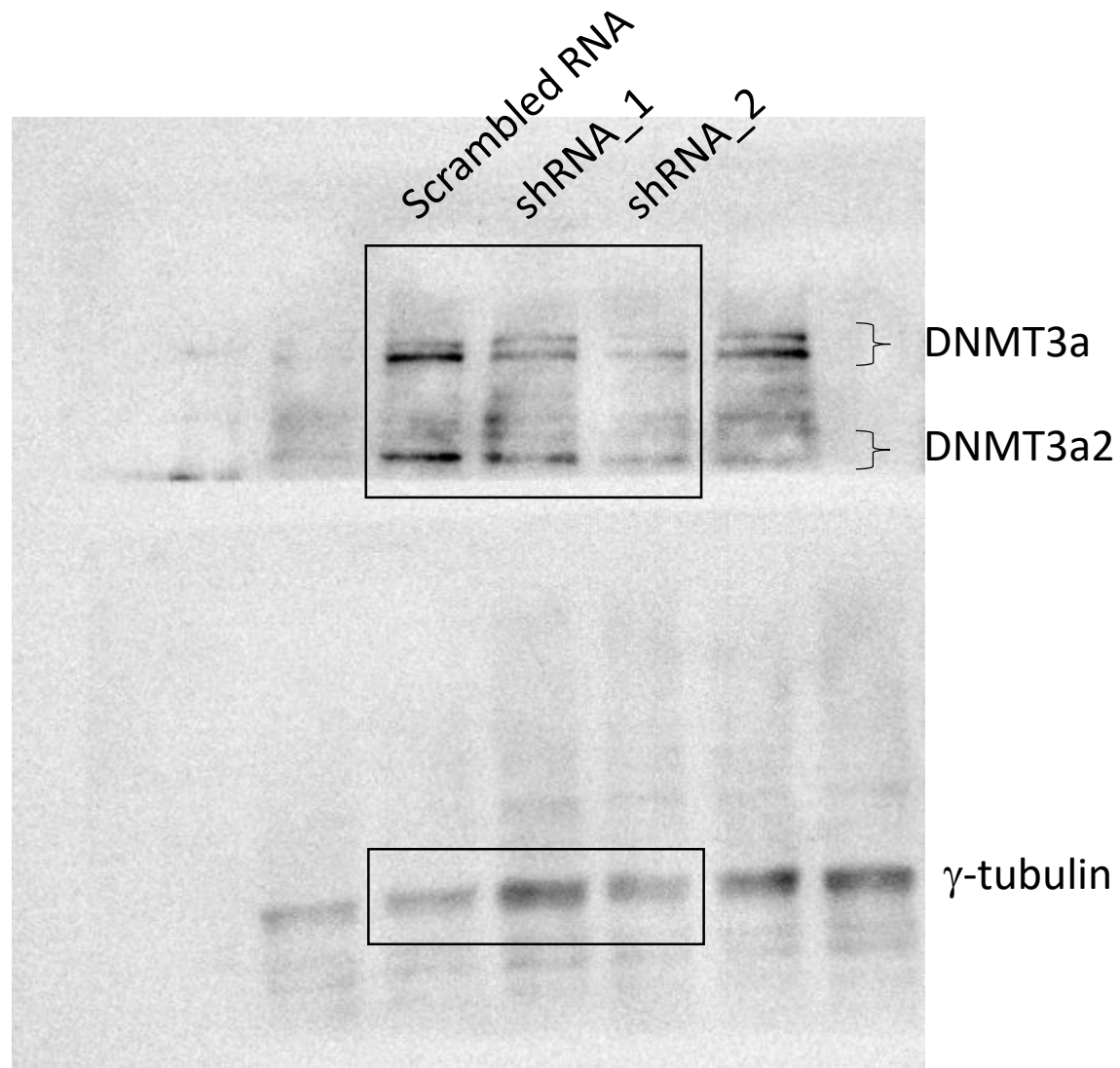

Supplement: S1 Raw Data — (ZIP) [file pbio.3001777.s007.zip › Supporting Information Raw Data/S3B_Fig.pdf]
